# Supplementary material for: An Engineered Thermostable Laccase with Great Ability to Decolorize and Detoxify Malachite Green
Source: Int J Mol Sci. 2021 Oct 29;22(21):11755. doi: 10.3390/ijms222111755 (PMC8583942; doi:10.3390/ijms222111755)
Supplement: Supplementary file 1 [file ijms-22-11755-s001.zip › ijms-1417109-supplementary.pdf]

Supplementary materials to

An Engineered Thermostable Laccase with Great Ability to Decolorize and Detoxify Malachite Green

Guotao Mao [1,2], Kai Wang [1], Fangyuan Wang [1], Hao Li [1], Hongsen Zhang [1,2], Hui Xie [1,2], Zhimin Wang [3], Fengqin Wang [1,2] and Andong Song [1,2,*]

1 Department of Microbiology, College of Life Sciences, Henan Agricultural University, Zhengzhou 450002, China; maoguotao@henau.edu.cn (G.M.); kaiwang@stu.henau.edu.cn (K.W.); wangfangyuan@ct-bio.com (Fangyuan Wang); haoli2021@stu.henau.edu.cn (H.L.); hszhang@henau.edu.cn (H.Z.); xiehuiliving421@vip.sina.com (H.X.); w_fengqin@henau.edu.cn (Fengqin Wang)
2 The Key Laboratory of Enzyme Engineering of Agricultural Microbiology, Ministry of Agriculture, Henan Agricultural University, Zhengzhou 450002, China
3 Department of Applied Chemistry, College of Science, Henan Agricultural University, Zhengzhou 450002, China; gary1451@iccas.ac.cn (Z.W.)
* Correspondence: songandong@henau.edu.cn

Table S1. Primers for site-directed mutagenesis of Mut2.

Primer	Sequence
H78A-F	ACAAGTTGCAGGCAACAACATTCTGG
H78A-R	TTGTTGCCTGCAACTTGTCGAACGGT
C119A-F	GCGGATGCATACCCGGTTCTGTTGG
C119A-R	CGGGTATGCATCCGCCACGAGGAC
H136A-F	CGGTGGCAGTTGGCTGGCGTGGTG
H136A-R	AGCCAACTGCCACCGCAGCAACGAC

The underlined bases indicate the mutated sites.

```
WT    1   MKMARNGKISYLEPEWITGSLRAGFTTRNGGISRAPYNSLNLGLNTEDPRPNVEGNRSTLVRAFGLEVHQ
Mut1  1   MKMARNGKIYYLEPEWITGSLRAGFTTRNGGISRAPYNSLNLGLNTGDPRPNVEGNRERLVRAFGLEPHQ
Mut2  1   MKMARNGKIYYLEPEWIHGSVRAGFTTRNGGISRPPYNSLNLGLNTGDPRPNVEGNRERLVRAFGLEPHQ
Mut3  1   MKMARQGKIYYLEPEWIHGSVRAGFTTRNGGISRPPYESLNLGLNTGDPRENVEGNRERLVRAFGLEPHQ

WT    71  LLTVRQVHGNNILVVDEPNPDLSHFQQVECDAIISNQPGMMIGVLVADCYPVLLAAPAQGVVAAVHVGWR
Mut1  71  LLTVRQVHGNNILVVDEPNPDLSHFQQVECDAIISNQPGMMIGVLVADCYPVLLAAPANGVVAAVHVGWR
Mut2  71  LLTVRQVHGNNILVVDEPNPDLSHFQQVECDAIISNQPGMMIGVLVADCYPVLLAAPANGVVAAVHVGWR
Mut3  71  LLTVRQVHGNNILVVDEPNPDLSHFQQVECDAIISNQPGMMIGVLVADCYPVLLAAPANGVVAAVHVGWR

WT    141 GAVAQIIQRTVDALQEQFGVCPEELLAAVGPGIGAHAYEVDRPVRDQFRKAGLPWAGVAEESRLGHWKLD
Mut1  141 GAVAQIIQRTVDALQEQFGVRPEDLLAAVGPGIGACCYEVDRPVRDQFRKAGLPWDEVAEESRLGHWKLD
Mut2  141 GAVAQIIQRTVDALQEQFGVRPEDLLAAVGPGIGACCYEVDRPVRDQFRKAGEPWDEVAEESRDGHWKLD
Mut3  141 GAVAQIIQRTVDAMQEQFGVRPEDLLAAVGPGIGACCYEVDRPVRDQFRKAGGPWDEVAEPSRDGHWKLD

WT    211 LRETCRRQLLTAGVVRTHIDLAEECTCCHRELLFSHRRDNGRTGRQLGFILLT
Mut1  211 LRETCRRQLLDAGVPREHIDLAEWCTCCHRELFFSHRRDNGRTGRQLGFILLT
Mut2  211 LREACRRQLLDAGVKAEHIDLAEWCTCCHRELFFSHRRDNGRTGRMLGFILLT
Mut3  211 LREACRRQLEDAGVKREHIYLAEWCTCCHRELFFSHRRDNGRTGRMLGFILLT
```

Figure S1. Sequence alignment of Ghlac WT, Mut1, Mut2, and Mut3. The mutated residues are colored red.

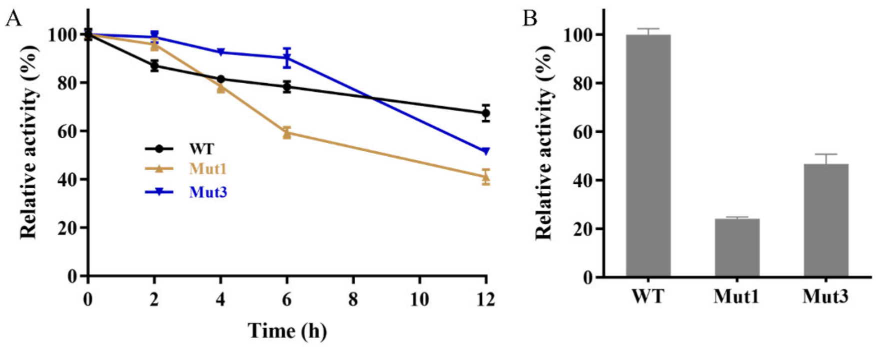

Figure S2. Thermostability at 50 °C (A) and activity against ABTS (B) of Mut1 and Mut3.

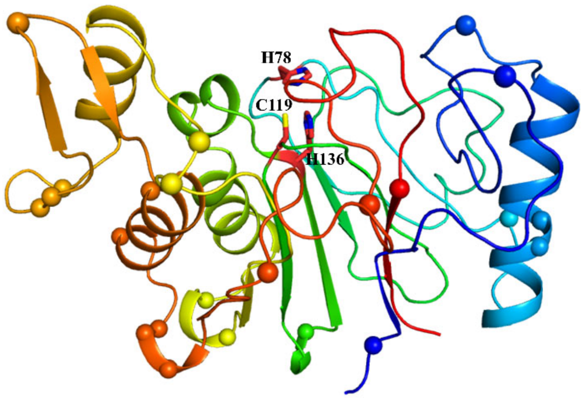

Figure S3. Structural mapping of the mutated residues of Ghlac Mut2. The mutated residues are shown as spheres. H78, C119, and H136 are labeled.

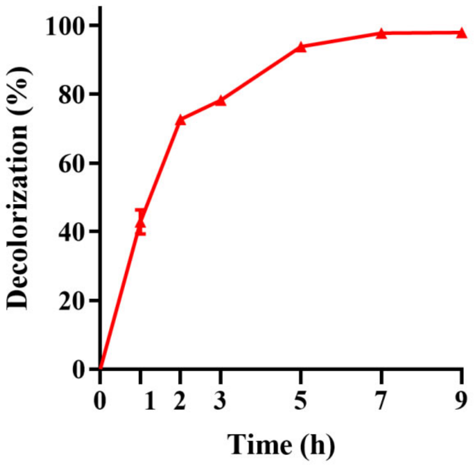

Figure S4. MG decolorization catalyzed by Ghlac WT. MG at the concentration of 100 mg/L was decolorized by 40 U/L of Ghlac WT with 0.1 mM ABTS at 70 °C.
